# A Custom DNA-Based NGS Panel for the Molecular Characterization of Patients With Diffuse Gliomas: Diagnostic and Therapeutic Applications

**DOI:** 10.3389/fonc.2022.861078

**Published:** 2022-03-17

**Authors:** Elena Tirrò, Michele Massimino, Giuseppe Broggi, Chiara Romano, Simone Minasi, Francesca Gianno, Manila Antonelli, Gianmarco Motta, Francesco Certo, Roberto Altieri, Livia Manzella, Rosario Caltabiano, Giuseppe Maria Vincenzo Barbagallo, Francesca Romana Buttarelli, Gaetano Magro, Felice Giangaspero, Paolo Vigneri

**Affiliations:** ^1^ Center of Experimental Oncology and Hematology Azienda Ospedaliero Universitaria (AOU) Policlinico “G. Rodolico - San Marco”, Catania, Italy; ^2^ Department of Surgical, Oncological and Stomatological Sciences, University of Palermo, Palermo, Italy; ^3^ Department of Clinical and Experimental Medicine, University of Catania, Catania, Italy; ^4^ Department of Medical and Surgical Sciences and Advanced Technologies “G.F. Ingrassia”, Anatomic Pathology, University of Catania, Catania, Italy; ^5^ Department of Radiological, Oncological and Anatomo-Pathological Sciences, La Sapienza University, Rome, Italy; ^6^ Department of Medical and Surgical Sciences and Advanced Technologies “G.F. Ingrassia”, Neurological Surgery, Policlinico “G. Rodolico - San Marco” University Hospital, University of Catania, Catania, Italy; ^7^ IRCCS Neuromed, Pozzilli, Italy

**Keywords:** glioma, next generation sequencing, biomarkers, molecular biology, diagnosis, targeted therapy, immunohistochemistry, fluorescence *in situ* hybridization

## Abstract

The management of patients with Central Nervous System (CNS) malignancies relies on the appropriate classification of these tumors. Recently, the World Health Organization (WHO) has published new criteria underlining the importance of an accurate molecular characterization of CNS malignancies, in order to integrate the information generated by histology. Next generation sequencing (NGS) allows single step sequencing of multiple genes, generating a comprehensive and specific mutational profile of the tumor tissue. We developed a custom NGS-based multi-gene panel (Glio-DNA panel) for the identification of the correct glioma oncotype and the detection of its essential molecular aberrations. Specifically, the Glio-DNA panel targets specific genetic and chromosomal alterations involving *ATRX chromatin remodeler (ATRX)*, *cyclin dependent kinase inhibitor 2A* (*CDKN2A)*, *isocitrate dehydrogenase* (NADP+) *1 (IDH1)* and the *telomerase reverse transcriptase (TERT)* promoter while also recognizing the co-deletion of 1p/19q, loss of chromosome 10 and gain of chromosome 7. Furthermore, the Glio-DNA panel also evaluates the methylation level of the *O-6-methylguanine-DNA methyltransferase* (*MGMT)* gene promoter that predicts temozolomide efficacy. As knowledge of the mutational landscape of each glioma is mandatory to define a personalized therapeutic strategy, the Glio-DNA panel also identifies alterations involving “druggable” or “actionable” genes. To test the specificity of our panel, we used two reference mutated DNAs verifying that NGS allele frequency measurement was highly accurate and sensitive. Subsequently, we performed a comparative analysis between conventional techniques - such as immunohistochemistry or fluorescence *in situ* hybridization - and NGS on 60 diffuse glioma samples that had been previously characterized. The comparison between conventional testing and NGS showed high concordance, suggesting that the Glio-DNA panel may replace multiple time-consuming tests. Finally, the identification of alterations involving different actionable genes matches glioma patients with potential targeted therapies available through clinical trials. In conclusion, our analysis demonstrates NGS efficacy in simultaneously detecting different genetic alterations useful for the diagnosis, prognosis and treatment of adult patients with diffuse glioma.

## Introduction

Diffuse gliomas are the most frequent primary brain tumors in the adult population accounting for more than 70% of all central nervous system (CNS) malignancies (1). They represent a heterogeneous group of tumors displaying different morphologic, genetic and epigenetic aberrations and an extremely variable response to therapy (2). Although diffuse gliomas comprise <1% of all newly diagnosed cancers, they are associated with significant morbidity and mortality (3).

The World Health organization (WHO) classification of CNS tumors has undergone major changes in 2016 and, subsequently, in 2021 (CNS 5 WHO). According to CNS WHO, a diagnosis of diffuse astrocytic or oligodendroglial gliomas must be based on histology but also on different molecular markers (4, 5).

Adult diffuse gliomas were usually characterized and classified by three major genetic events: **i]** mutations in *isocitrate dehydrogenase* (NADP+) *1* (*IDH1*) or *isocitrate dehydrogenase* (NADP+) *2* (*IDH2*) genes, **ii]** detection of the 1p/19q chromosomal co-deletion and **iii]** nuclear retention or loss of the *ATRX chromatin remodeler (ATRX*) (6). On the basis of the 2021 updated classification, additional molecular biomarkers have become essential to categorize adult gliomas including: *H3.3 Histone A* (*H3.3A*) mutations for diffuse midline gliomas*, telomerase reverse transcriptase (TERT*) promoter mutations, *epidermal growth factor receptor* (*EGFR*) gene amplification and chromosome 7 gain combined with loss of chromosome 10 for glioblastoma (GBM), and homozygous deletions of both *cyclin dependent kinase inhibitor 2A* (*CDKN2A*) and *cyclin dependent kinase inhibitor 2B* (*CDKN2B*) loci for *IDH*-mutant astrocytoma (7). In addition, the detection of *tumor protein p53* (*TP53*) mutations (linked to an inferior prognosis and lower response to chemotherapy) (8), or the assessment of *O-6-methylguanine-DNA methyltransferase* (*MGMT*) promoter methylation status (useful to guide the use of alkylating agents), represent further molecular biomarkers deserving of careful investigation in diffuse low or high grade gliomas (9).

A genomic landscape investigation of GBM samples carried out by The Cancer Genome Atlas (TCGA) consortium in over 500 tumor samples revealed that the aberrant activation of PI3K/AKT, TP53 or RB signaling pathways are correlated with treatment response or survival (10). It has also been reported that genetic alterations, such as loss or mutations in the *phosphate and tensin homologue* (*PTEN*) gene or overexpression of the *platelet-derived growth factor receptor alpha* (*PDGFRA*) gene, represent additional molecular biomarkers that may be correlated with tumor evolution and poor prognosis (11, 12). Finally, different receptor tyrosine kinases (RTKs) and their ligands represent promising therapeutic targets for the treatment of GBM because they are involved in disease invasiveness, angiogenesis and cancer cell survival (13, 14).

Since the diagnosis and treatment of malignant brain tumors represents one of the most challenging problems in clinical oncology, the use of novel techniques such as next generation sequencing (NGS) embodies a versatile solution for the simultaneous analysis of different genes associated with tumor development, progression, and resistance to therapy (15–18). Indeed, NGS interrogates multiple genes and target variants simultaneously, employing a small amount of tumor sample (19) and can identify single nucleotide variants (SNVs), short insertions or deletion mutations (indel) and large regions of loss of heterozygosis (LOH) (20).

Aim of this study was to investigate the efficacy of a custom NGS panel, called Glio-DNA panel, to guide diagnosis and molecular characterization of low and high-grade gliomas, through the simultaneous detection of the main mutations and copy number alterations (CNAs) occurring in these tumors. Furthermore, we investigated the clinical usefulness of this panel in the detection of alterations involving druggable and actionable genes, in order to define potential therapeutic approaches for glioma patients.

## Materials and Methods

### Clinical Samples Collection

Glioma samples were collected from 60 patients surgically treated in the Neurosurgery Unit of the A.O.U. Policlinico “G. Rodolico - San Marco”, Catania - Italy, between January 2015 and July 2021. All tumors were classified following the 2021 CNS WHO guidelines (5). Our cohort included: 44 GBMs, 5 astrocytomas, 5 oligodendrogliomas, 4 gangliogliomas, 1 gliosarcoma (GS) and 1 diffuse midline glioma. Clinical and pathological data were retrospectively retrieved from institutional medical records. Formalin-fixed paraffin-embedded (FFPE) tumor tissues of archival glioma cases were obtained from the Pathology Department “G.F. Ingrassia” of the University of Catania - Italy. The study was approved by the local ethics committee (protocol code 166/2015/PO). All patients signed a specific informed consent before surgery in accordance with the Declaration of Helsinki. Data were anonymized before analysis to protect the patients’ identity.

### DNA Extraction, Bisulfite Conversion and Quantification

For each specimen, 5-micron thick sections were cut from FFPE blocks using a standard microtome. Tumor content was determined with hematoxylin and eosin stained slides by a pathologist. DNA extraction and molecular analyses were performed at the Center for Experimental Oncology and Hematology of A.O.U. Policlinico “G. Rodolico - San Marco” in Catania, Italy. For genomic DNA extraction, the tumor area was macro-dissected from unstained slides with a sterile scalpel and processed using the QS Gene Read FFPE Treatment kit and QIAsymphony DNA Mini Kit (both from Qiagen) employing the automated QIAsymphony instrument according to the manufacturer’s instruction. DNA concentration was measured with the Qubit 3.0 fluorometer (ThermoFisher Scientific), using the dsDNA HS Assay kit (ThermoFisher Scientific). For the *MGMT* promoter methylation analysis, bisulfite modification was performed on 100 ng of genomic DNA using the EpiTech Bisulfite kit (Qiagen) according to the manufacturer’s instructions. Single-stranded bisulfite converted DNA was quantified using the Qubit ssDNA Assay kit (ThermoFisher Scientific) on a Qubit 3.0 fluorometer.

### Next Generation Sequencing Panel Design and Library Preparation

Libraries were prepared using a custom primer panel designed using the Ion AmpliSeq Designer tool (https://www.ampliseq.com/login/login.action). The panel encompasses 2361 amplicons in 3 primer pools covering the coding DNA sequencing (CDS) of 65 genes associated with the diagnosis and potential treatment response of glioma patients (**Supplementary Table 1**). In addition, 70 primer pairs for the analysis of specific single nucleotide polymorphisms (SNPs) were added in order to detect the loss of heterozygosity (LOH) of chromosomes 1p, 19q, 7 and 10, as previously reported (21). DNA libraries were generated with the Ion AmpliSeq library kit plus (ThermoFisher Scientific) using 30 nanograms of genomic DNA (10 nanograms per primer pool). Libraries were barcoded employing the Ion Xpress barcode adapter kit (ThermoFisher Scientific).

The *TERT* promoter and *MGMT* promoter libraries were prepared using the Ion Plus Fragment Library kit (ThermoFisher Scientific) according to the manufacturer’s instructions containing specific details about library preparation without fragmentation. Specifically, for the amplification of the *TERT* promoter, 40 ng of genomic DNA were amplified with the Platinum PCR SuperMix High Fidelity (ThermoFisher Scientific) using the following forward 5’-TTCCCACGTGCGCAGCAG-3’ and reverse 5’-GCTCCCAGTGGATTCGCG-3’ primers. For the amplification of the *MGMT* promoter, 70 ng of bisulfite treated genomic DNA were used, employing the Phusion U Hot Start PCR master mix (ThermoFisher Scientific) and the following forward 5’-TTTCGGATATGTTGGGATAG-3’ and reverse 5’-GATTTGGTGAGTGTTTGGGT-3’converted primers.

All libraries were then quantified by qPCR with the Ion Library TaqMan Quantitation kit (ThermoFisher Scientific) employing the ABI 7500 Real-Time PCR System (Applied Biosystem) and diluted to equimolar amounts before pooling. Manual template preparation and enrichment were performed employing the Ion PGM HiQ OT2 kit on the Ion OneTouch 2 system and the Ion One Touch ES instrument (all from ThermoFisher Scientific). Enriched libraries were then loaded on the Ion 318 v2 BC chip and massive parallel sequencing was carried out using the Ion PGM HiQ Sequencing kit on the Ion Torrent Personal Genome Machine (Ion PGM) platform according to the manufacture’s instruction (all from Thermo Fisher Scientific).

### Next Generation Sequencing Data Analysis

Sequencing raw data were aligned to the hg19 (GRCh37) reference genome. The Ion PGM Torrent Suite v.5.8.0 (ThermoFisher Scientific) was employed to perform initial quality control including chip loading density, median read length and number of mapped reads. Ion Reporter v5.12.0 (ThermoFisher Scientific) was used for SNV annotation. Variants were filtered considering a cut off of 3% for variant allele frequency (VAF), read depth >100, a Phred quality score >40 and a p-value <0.0001 in order to exclude false positive variants. Variants were subsequently annotated against the Cancer Mutation Census (CMC) version 94 database (22) and only mutations categorized as tier 1, 2, and 3 were considered pathogenic.

To identify somatic copy number alterations (CNAs), a reference baseline consisting of ten male subjects not displaying CNAs was created on the Ion Reporter software. The call of a CNA was made for samples showing a MAPD (Median of the Absolute values of all Pairwise Differences) <0.45, a metric that evaluates whether panel data can be used for CNA analysis and filtered to exclude regions with confidence lower that 30.

To evaluate the methylation status of the *MGMT* promoter, the detection call rate for four CpG islands (5’-CGACGCCCGCAGGTCCTCG-3’, underlined bases) was determined using the Torrent Suite Variant Caller plugin. An average methylation percentage below 10% was considered unmethylated, between 10 and 50% was scored as moderately methylated and above 50% highly methylated. Bisulfite conversion efficiency was evaluated by examining cytosine to thymine conversion for cytosines not in CpG motifs. Variant calling to assess *TERT* promoter mutations was also performed using the above indicated plugin.

### Analytical Validation of the Glio-DNA Panel

The initial performance of the Glio-DNA panel was evaluated using two reference standard DNA samples purchased from Horizon Discovery. OncoSpan gDNA Reference Standard (catalog ID HD827) and OncoSpan FFPE Reference Standard (catalog ID HD832) were used as positive controls for variant calling. OncoSpan gDNA is a reference standard harboring 386 variants in 152 genes while OncoSpan FFPE contains over 380 variants across 152 key cancer genes.

### Sanger Sequencing

Hot-spot mutation in *IDH1*, the *TERT* and *MGMT* promoters were analyzed by direct Sanger sequencing after PCR-based amplification of the specific locus. Fifty nanograms of genomic DNA were amplified with the Platinum PCR SuperMix High Fidelity following the manufacturer’s instructions. The hot-spot mutation in *IDH1* was detected with the following forward 5’-AAGTTGAAACAAATGTGGAAATCACCAAA-3’ and reverse 5’-CCAACATGACTTACTTGATCCCCATA-3’ primers. PCR-based amplification of *TERT* and *MGMT* promoters were performed using the previously described primer pairs. The obtained PCR products were then resolved on agarose gel electrophoresis, purified and sequenced.

To evaluate the percentage of the *MGMT* promoter methylation, the ratio of cytosine to thymine at each specific CpG site was determined. The CpG sites were classified as ‘methylated’ if the peak in the cytosine/thymine ratio was >20%, or were scored as unmethylated if the ratio was <20%. An average methylation percentage was then calculated.

### Immunohistochemistry

Each tumor underwent immunohistochemistry, as previously described (23, 24), using the standard streptavidin-biotin-labeling system. Briefly, deparaffinized tissue sections were incubated with antibodies against p53 (mouse monoclonal, clone DO-7, Santa Cruz Biotechnology, Dallas, TX, USA), ATRX (mouse monoclonal, clone AX1; Dianova), IDH1 R132H (mouse monoclonal, clone H09, Dianova) and H3.3 K27M (rabbit monoclonal, clone RM-192, ThermoFisher Scientific). Immunohistochemical staining for p53, ATRX and H3.3 K27M were considered as positive if brown chromogen was present within the cell nuclei, while cytoplasmic reactivity for IDH1 R132H was interpreted as positive.

### Fluorescence *In Situ* Hybridization

Five-micron sections were cut from FFPE samples for fluorescence *in situ* hybridization (FISH) analysis with Leica Biosystems Tissue Digestion Kit (Leica Biosystems). Slides were deparaffinized in an oven at 70°C for 60 minutes and exposed to xylene for 10 minutes, dehydrated in ethanol and treated with pretreatment solution (1 mol/L sodium thiocyanate) for 30 minutes at 80°C; samples were then digested in pepsin solution (0,65% in protease buffer) for 30 minutes, washed twice in SSC1x buffer, and air-dried. The mix for each probe (5 to 15 μl) was added to each slide according to the manufacturer’s instructions (Abbott Molecular). The analysis of 1p/19q co-deletion status was performed using the Vysis LSI 1p36/1q25 and 19q13/19p13 Dual-Color Probe kit (04N60-020). Chromosome 10 loss was analyzed using the Vysis LSI PTEN (10q23)/CEP 10 FISH Probe Kit (04N62-020). The determination of chromosome 7 gain and *EGFR* amplification were both analyzed with LSI EGFR (7p11)/CEP 7 probe set (01N35-020). *CDKN2A* homozygous loss was assessed using the Vysis LSI CDKN2A (9p21)/CEP 9 Probe kit (04N61-020).

Target DNA and probes were co-denatured at 80°C for 5 minutes and incubated at 37°C overnight in a humidified hybridization chamber (ThermoBrite TopBrite). Post-hybridization washes were performed in wash solution at 74°C for 2 minutes. Sections were finally counterstained with DAPI (4′, 6-diamidino-2-phenylindole), cover slipped, and stored in the dark prior to microscope analysis.

### Fluorescence *In Situ* Hybridization Interpretation

FISH sections were examined with an Axio Imager M1 microscope (Carl Zeiss) equipped with Z-stack and appropriate filters by two independent investigators (SM, FRB). The most representative areas of each tumor were previously selected with H&E staining by expert pathologists. Signals were counted in at least 200 neoplastic nuclei for each sample, and images were captured using a Metasystem station (Zeiss MetaSystems) with sequential DAPI, FITC, and rhodamine filter settings; the resulting images were automatically reconstituted with blue, green, and orange colors by the software. For all analyzed probe kits, orange (O) and green (G) signals were enumerated under the fluorescence microscope and then re-counted in the acquired images. Ratio was calculated by dividing the number of LSI orange signals by the number of green reference signals, according to published methods. Samples were considered positive for 1p/19q co-deletion loss when ≥30% of neoplastic nuclei exhibited 1O/2G signals for both 1p36/1q25 and 19q13/19p13, considering a ratio ≤0.70 as allelic loss (25, 26).

Regarding *EGFR* amplification, a signal ratio between 1-2 was considered as gain, while a ratio >2 (5O/2G) was considered as amplification; in particular, specimens were considered amplified for *EGFR* when more than 10% of tumor cells exhibited either a ratio >2 or innumerable clusters of orange LSI signals (27). For whole-chromosome 7 gain and whole-chromosome 10, samples were considered positive when ≥30% of neoplastic cells showed 3O/3G signals (gain of chromosome 7) and 1O/1G signals (loss of chromosome 10), respectively. However, the limitation of FISH analysis for entire chromosome alterations should be considered.

Finally, samples were evaluated as positive for *CDKN2A* homozygous deletion when ≥20% of tumor cells exhibited the absence of both orange signals in the presence of at least 1 reference green signal (0O/1-2G) (27, 28).

### Statistical Analysis

Simple linear regression analysis was used to analyze the correlation between expected and measured mutation allele frequencies of the reference standard. Statistical analysis was carried out using the GraphPad Prism v 5.0a software. Cohen’s κ was calculated to assess the consistency of immunohistochemistry staining, Sanger sequencing or FISH versus NGS. Cohen’s κ <0.4 was considered weak, ≥0.4 but <0.8 was considered moderate and ≥0.8 was considered strong. Statistical analysis was performed with the GraphPadQuickCalc software (https://www.graphpad.com/quickcalcs/index.cfm). Sensitivity was calculated according to the formula [true positive/(true positive + false negative)] × 100, while the specificity was determined employing the formula [true negative/(true negative + false positive)] × 100.

## Results

### Glio-DNA Panel Sequencing Performance

**Figure 1:**
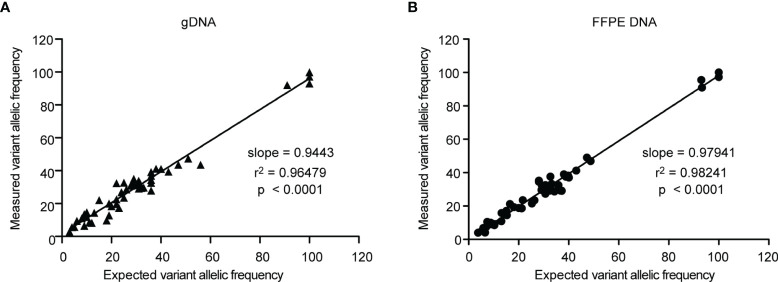
Linear regression analysis of the expected allele frequencies versus measured allele frequencies employing the Glio-DNA panel. Correlation between expected and measured variant allele frequencies (expressed as percentage) evaluated by regression analysis for genomic (left panel) and FFPE (right panel) standard DNA using the Glio-DNA panel.

To assess the accuracy of the Glio-DNA panel, we employed a cell-line derived DNA reference harboring mutations in 26 of the 66 genes included in our panel. Moreover, in order to reproduce the extraction method employed for our glioma samples, we also employed an FFPE DNA reference harboring the same variants. Both DNA references were sequenced twice to compare variant detection and their VAF. Overall, we detected 53 alterations with a VAF >2% out of the 55 different gene alterations (**Supplementary Tables 2, 3**). As expected, two mutations in the *EGFR* gene went undetected as their allele frequency (AF) was below our pre-established limit of detection. We also evaluated the correlation between the expected and measured AF by regression analysis. The slope of the regression line for genomic DNA was 0.9443 with an r^2^ of 0.96479 (**Figure 1A**), while FFPE-extracted DNA exhibited a regression line of 0.97941 with an r^2^ of 0.9824 (**Figure 1B**). These results indicate that AF measurements with the Glio-DNA panel were highly accurate with a panel sensitivity of 96.1%.

### Comparative Analysis Between Conventional Molecular Testing and the Glio-DNA Panel

The use of different diagnostic assays as part of routine clinical testing for the 60 glioma specimens provided us an opportunity to compare NGS performance with those of more conventional methodologies such as immunohistochemistry (IHC), Sanger sequencing and FISH. The concordance data between conventional testing and NGS are reported in **Table 1**. In this analysis we included five gliomas WHO grade 2 (namely three oligodendrogliomas, one astrocytoma and one ganglioglioma) and 55 WHO grade 3 or 4 gliomas. The latter included: two oligodendrogliomas, four astrocytomas, three gangliogliomas, one GS, one diffuse midline glioma and forty-four GBMs.

**Table 1:** Concordance between conventional testing and NGS.

Variable	IDH1 R132H (IHC)	ATRX loss (IHC)	1p/19q codeletion (FISH)	CDKN2A/B loss (FISH)	TERT promoter mutation (Sanger)	EGFR amplification (FISH)	Chr7 imbalance (FISH)	Chr10 loss (FISH)	TP53 mutation (IHC)
by CT N° samples analysed	60	60	10	18	45	39	33	33	59
Positive for both	7	3	5	5	33	4	19	20	15
Positive for CT and Negative for NGS	0	1	0	0	0	1	0	0	3
Positive for NGS and Negative for CT	1	0	0	0	0	0	0	0	2
Positive for neither	52	56	5	13	12	34	14	13	39
Cohen’s k	0.923	0.848	1	1	1	0.874	1	1	0.800
Sensitivity for CT, %	87.50	100.00	100.00	100.00	100.00	100.00	100.00	100.00	88.24
Specificity for CT, %	100.00	98.25	100.00	100.00	100.00	97.14	100.00	100.00	92.86
Sensitivity for NGS, %	100.00	75.00	100.00	100.00	100.00	80.00	100.00	100.00	83.33
Specificity for NGS, %	98.11	100.00	100.00	100.00	100.00	100.00	100.00	100.00	95.12

CT, conventional testing; NGS, next generation sequencing; IHC, immunohistochemistry; FISH, fluorescence in situ hybridization.

According to the CNS 5 WHO classification and cIMPACT-NOW recommendations, identification of multiple molecular biomarkers is pivotal for the correct categorization of diffuse gliomas, especially in the adult population (29, 30).

#### IDH Mutations

**Figure 2:**
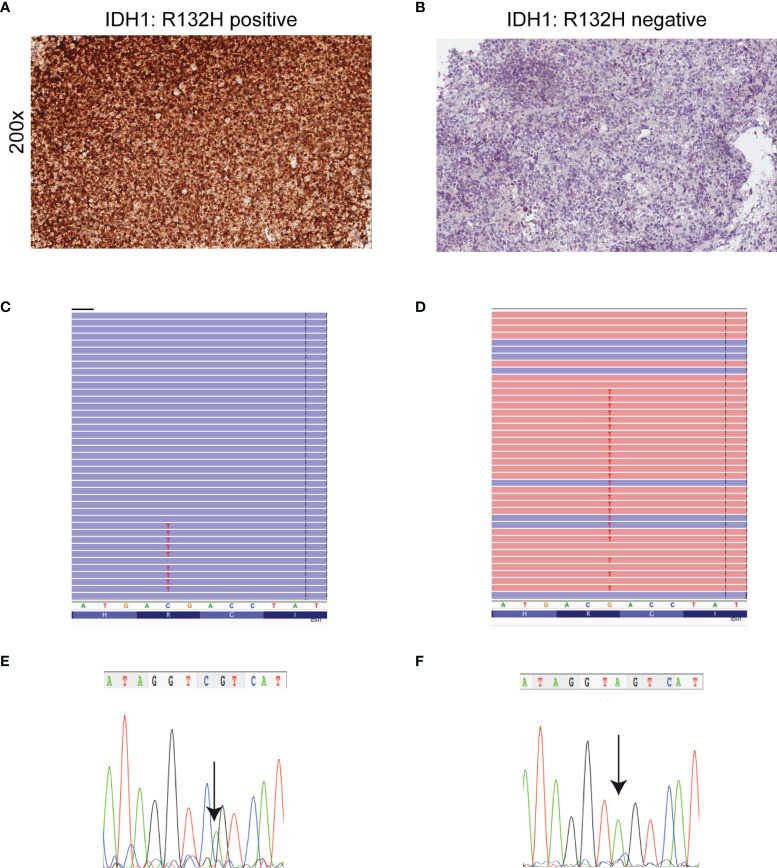
Detection of mutant IDH1 by immunohistochemistry, NGS and Sanger sequencing. **(A)** Astrocytoma cells are diffusely and strongly stained with anti-IDH1 (R132H) antibody (immunoperoxidase; original magnification 200x). **(B)** Corresponding *IDH1* c.395G>A (p. R132H) mutation detected by NGS. Results were viewed in the Integrative Genomics Viewer (IGV). **(C)** The chromatogram showing representative sequencing results of *IDH1* c.395G>A (p. R132H). **(D)** Absence of IDH1 (R132H) immunoreactivity in a *IDH1*- (R132S)-mutant astrocytoma. **(E)** *IDH1* c.394C>A (p. R132S) mutation detected by NGS. Results were viewed in the IGV. **(F)** The chromatogram showing the representative sequencing results of IDH1 c.394C>A. Note that *IDH1* is a negative-sense gene with respect to the genomic reference sequence. Thus, any nucleotide change is displayed as reverse complement. The arrow symbols on chromatogram indicate the place of mutation.

When we compared the NGS results obtained for *IDH1* with IDH1-R132H IHC conventional testing we found that IHC analysis was concordant with NGS results (**Figures 2A** **, C**), with the exception of one case (case 49) (**Table 1** and **Supplementary Table 4**). Interestingly, this astrocytoma was negative for IDH1-R132H expression by IHC (**Figure 2B**) analysis but NGS showed the non-canonical variant IDH1-R132S (**Figure 2D**) usually associated with a more favorable clinical outcome (31). Sanger sequencing was used to confirm NGS results for investigated variants (**Figures 2E, F**).

#### Loss of Nuclear ATRX

**Figure 3:**
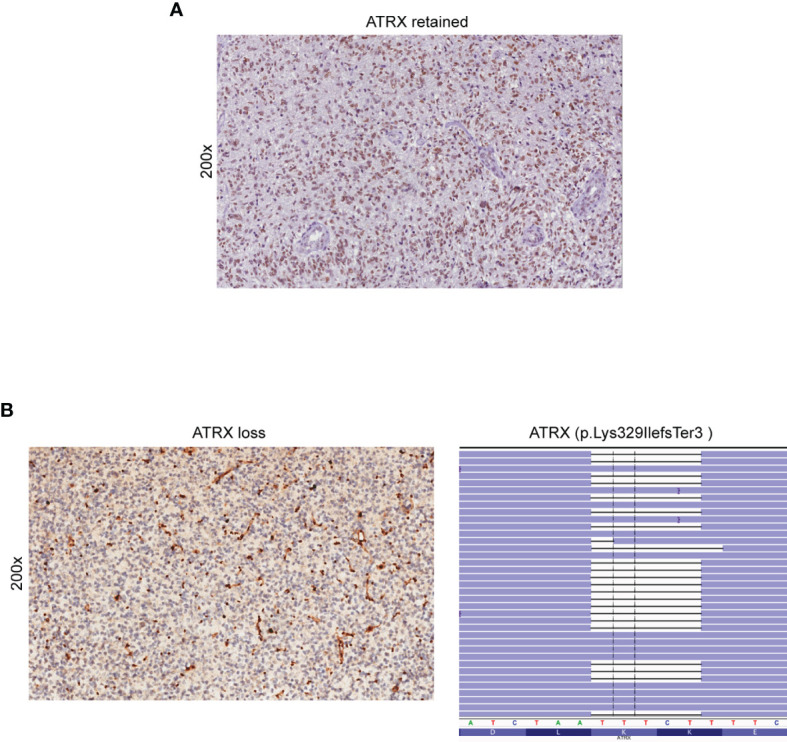
Detection of ATRX loss by immunohistochemistry and NGS. **(A)** Retained nuclear expression of ATRX in neoplastic glioma cells (immunoperoxidase; original magnification 200x). **(B)** Loss of nuclear expression of ATRX in a sample of astrocytoma (case 49) (left panel). Note the retained immunoreactivity in endothelial cells and non-neoplastic entrapped glial cells (immunoperoxidase; original magnification 200x). NGS demonstrated the presence of the ATRX p. Lys329IlefsTer3 truncated mutation (case 49) (right panel). Results were viewed in the IGV.

The lack of nuclear staining by IHC usually corresponds to ATRX loss of function alterations, mostly missense or truncating mutations (32). IHC- and NGS-based testing for ATRX loss was concordant in 59 out of 60 glioma cases. Three cases harboring an *ATRX* truncating mutation detected by NGS (case 37 - p.Lys698Ter, case 49 - p.Lys329IlefsTer3 and case 54 - p.Ala1988ValfsTer27) showed negative staining by IHC, indicating loss of ATRX nuclear expression (**Figure 3**). However, a discrepancy was observed in the analysis of case 33 as IHC indicated loss of nuclear ATRX while NGS failed to detect pathogenic *ATRX* mutations.

#### 1p/19q Co-Deletion

**Figure 4:**
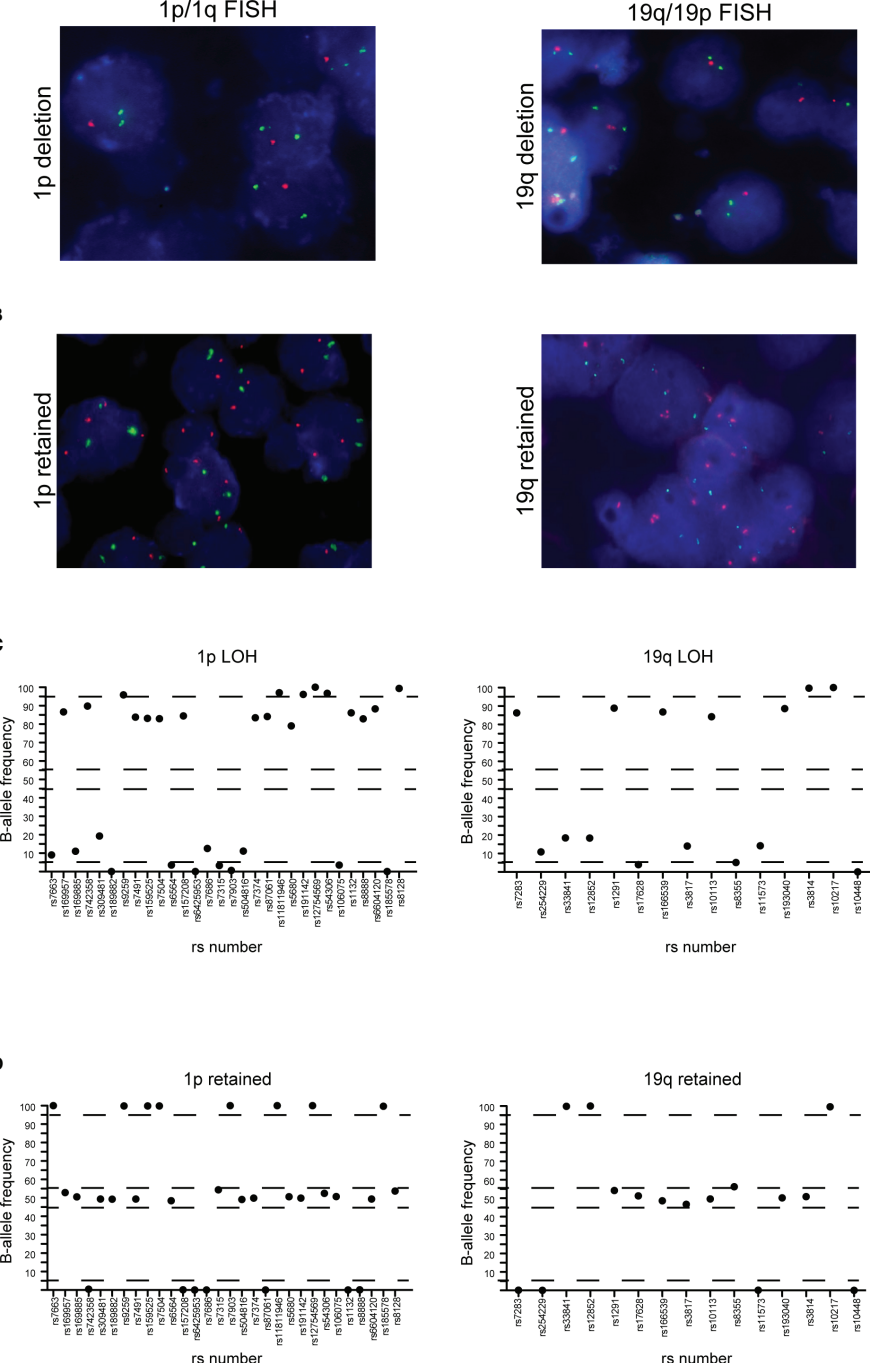
Correlation between FISH and NGS detection of 1p/19q co-deletion. **(A)** 1p (left panel) and 19q (right panel) co-deletion in a case of oligodendroglioma detected by FISH. **(B)** 1p (left panel) and 19q (right panel) negative control. In both cases, red dots represent 1p36.3 and 19q13.3 signals while green dots represent 1q25.2 and 19p13.2 signals. **(C)** Distribution of B-allele frequencies based on loss of heterozygosis (LOH) of single nucleotide polymorphisms (SNPs) in NGS of an oligodendroglioma sample with typical 1p (left panel) and 19q (right panel) co-deletion. **(D)** GBM without LOH and genomic alterations of chromosome 1p (left panel) and 19q (right panel). The x axis shows the investigated SNPs (rs number was reported) while the y axis shows the percentage variant (B-allele frequency). Dashed lines indicate the arbitrarily set homozygosis range of 0-5% and 95-100%. The range of heterozygosis is defined as being between 45% and 60% of the B-allele frequency. LOH was called when the B-allele frequency of a SNP was outside the established range for homo- and heterozygosis.

To detect the 1p/19q co-deletion in our glioma samples, we employed SNP-based loss of heterozygosis (LOH) analysis by NGS, as previously reported (21, 33). During the design of the Glio-DNA panel, we included 45 highly polymorphic SNPs located on the short arm of chromosome 1 (1p) and on the long arm of chromosome 19 (19q) and evaluated the allelic imbalance in our patient cohort. In all five oligodendroglioma cases (cases 19, 27, 32, 38 and 41), NGS analysis provided the same 1p/19q results obtained by FISH (**Figure 4** and **Table 1**). Furthermore, these cases harbored an activating *IDH1* mutation supporting the histopathological diagnosis of oligodendroglioma. Surprisingly, NGS also allowed the identification of a rare case of GBM (case 2) displaying the 1p/19q co-deletion (**Supplementary Table 4**). Lastly, SNP-based LOH by NGS identified 8 cases (13.3%) of GBM (cases 7, 12, 13, 22, 24, 29, 31 and 34) with an allelic imbalance involving only chromosome 19q while no case showing an allelic loss of chromosome 1p was detected.

#### Deletion of CDKN2A/CDKN2B

We analyzed *CDKN2A/CDKN2B* by NGS in 45 of 60 specimens included in our cohort. We found that 5 patients (11.6% of total tested samples) showed a biallelic loss of *CDKN2A/CDKN2B* (homozygous deletion) while 5 (11.6%) displayed hemizygous deletions of these two genes. The presence of *CDKN2A/CDKN2B* biallelic and monoallelic loss was tested employing FISH in 18 tumor samples with no discrepancies observed when compared to NGS (**Supplementary Figure 1**).

#### TERT Promoter Mutations

Since the TERT promoter region is difficult to amplify because of its high guanine-cytosine (GC) content (>80%), we designed primers to separately amplify the region of interest and subsequently added the obtained amplicon to the Glio-DNA panel. NGS analysis detected *TERT* promoter mutations in 33 out of 45 investigated gliomas (72.7%), 12 of which harbored the hot-spot C250T (c.1-146C>T) mutation and 21 carrying the C228T (c.1-124C>T) variant. The NGS results were concordant with those acquired by Sanger sequencing as confirmed by specificity and sensitivity values (**Table 1**).

#### EGFR Amplification

**Figure 5:**
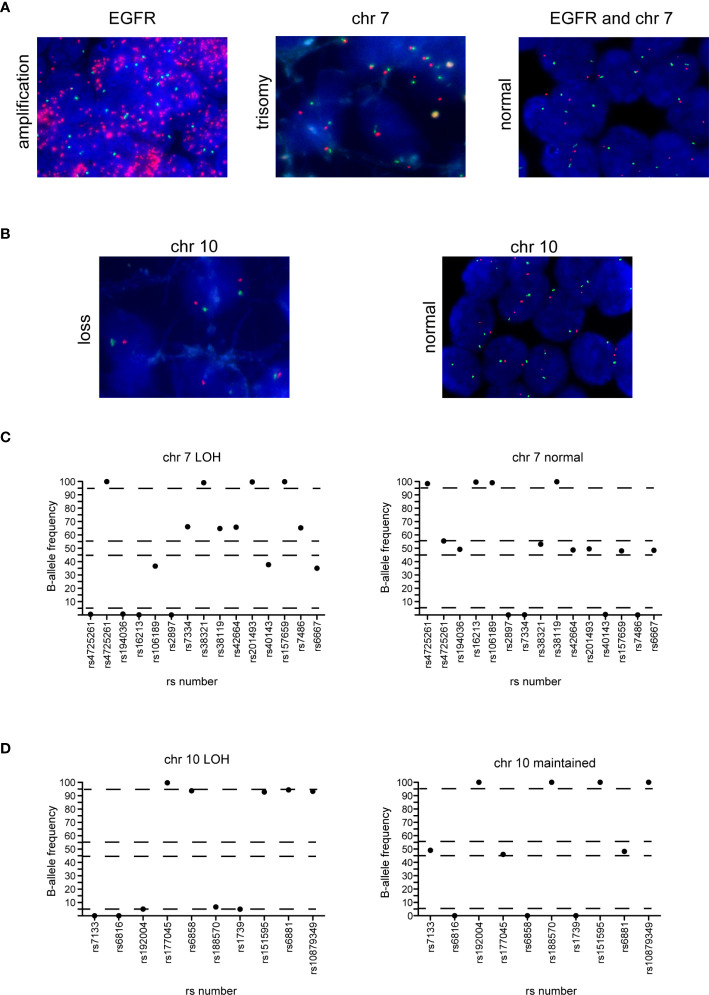
Correlation between FISH and NGS detection of EGFR amplification, chromosome 7 gain and chromosome 10 loss. **(A)** EGFR amplification (left panel) and trisomy of chromosome 7 (middle panel) in two cases of GBM and normal EGFR expression and chromosome 7 (right panel) in a case of astrocytoma detected by FISH. Red dots represent the *EGFR* gene while green dots represent the centromere of chromosome 7. **(B)** Chromosome 10 loss (left panel) and negative control (right panel) in two glioma samples. Red dots represent 10q23 signal while green dots represent the CEP 10 signal. **(C)** Distribution of B-allele frequencies based on LOH of SNPs of a GBM sample with typical chromosome 7 gain (left panel) and an astrocytoma sample without chromosome 7 gain (right panel). **(D)** Distribution of B-allele frequencies based on LOH of a GBM sample showing chromosome 10 loss (left panel) and a sample without LOH of chromosome 10 (right panel). The x axis shows the investigated SNPs (rs number was reported) and the y axis shows the percentage variant (B-allele frequency). Dashed lines indicate the arbitrarily set homozygosis range of 0 - 5% and 95 - 100%. The range of heterozygosis is defined as being between 45% and 60% of B-allele frequency. LOH was called as indicated in the legend of **Figure 4**.

The Glio-DNA panel was designed to identify SNV, but also to detect copy number alterations (CNAs) for all genes included in the panel. *EGFR* CNAs were evaluated in 39 of 60 glioma samples with a MAPD score <0.45. We found concordance in 38 of 39 cases (97% - 4 amplified and 34 negative cases) with informative NGS results (**Table 1** and **Figure 5A**).

#### +7/-10 Cytogenetic Signature

To detect the large genomic aberrations involving chromosome 7 (harboring *BRAF*, *CDK6*, *EGFR* and *MET*) and 10 (that includes *FGFR2*, *MGMT* and *PTEN*), during the design of the Glio-DNA panel we included 25 highly polymorphic SNPs spanning chromosome 7 and 10 (34, 35). We found that FISH- and NGS-based testing for chromosome 7 gain was concordant in all analyzed cases with informative FISH results (n=33) (**Table 1** and **Figures 5A, C**). In detail, we found that 16 out of 22 GBM cases (72.7%) showed an imbalance/gain of chromosome 7 as well as two astrocytomas (cases 30 and 49) and one ganglioglioma (case 48). Moreover, no discrepancies were observed between FISH and NGS among the 33 gliomas analyzed for chromosome 10 loss (**Figures 5B, D**). We also detected a high concordance between the co-presence of chromosome 7 gain and 10 loss (90.9%, 30 out 33 samples).

#### BRAF V600E and H3-3A K27M Mutations

In our cohort, NGS sequencing detected the V600E (c.1799T>A) substitution in a case of ganglioglioma grade 1 (case 39). Since the detected VAF was very low (2.5%) this would explain why IHC analysis failed to identify this mutation.

A single case of diffuse midline glioma (case 37) was included in our cohort. Diffuse midline gliomas are located in the midline brain structures and are characterized by the presence of a lysine-to-methionine mutation at amino acid 27 of histone H3.3 encoded by the *H3-3A* gene (36). In this case we found a perfect concordance between IHC- and NGS-based testing as both identified H3-3A p.K27M (**Supplementary Figure 2A**).

#### TP53 Alterations

We detected *TP53* missense mutations resulting in TP53 protein overexpression in 15 cases, and a non-sense *TP53* mutation resulting in lack of TP53 immunostaining in one sample (case 42). Of note, in two cases (31 and 57), TP53 immunostaining suggested the presence of a mutant TP53, while NGS analysis identified wild-type *TP53*. On the contrary, in three specimens (cases 42, 48 and 54) NGS analysis detected the presence of pathogenic *TP53* mutations (p.Trp146Ter, p.Arg273His and p.Arg267Trp, respectively) while IHC did not show TP53 immunostaining. Although in five cases there was a discordance between NGS and IHC, the sensitivity and specificity for each assay was always >80% (**Table 1** and **Supplementary Figures 2B, C**).

#### MGMT Promoter Methylation

Although *MGMT* promoter methylation has limited diagnostic value, it is of great importance to guide treatment decisions on the use of chemotherapy with alkylating agents for patients with *IDH1*-wild-type gliomas and GBMs (37). Different assays can be employed to investigate the methylation status of the *MGMT* promoter, such as pyrosequencing, methylation-specific PCR, methylation arrays or Sanger sequencing (38). We tested the possibility to employ NGS to measure methylation levels in four cytosine-phosphate-guanine (CpG) islands located in exon 1 of the *MGMT* gene (chr10: 131,265,519 to 131,265,537; GRCh37-hg19). Libraries from a single amplicon pool were obtained and sequenced for 57 out of 60 glioma samples. We identified 9 samples (15.8%) scored as unmethylated, 14 samples (24.6%) expressing moderate methylation levels and 35 samples (61.4%) with high methylation (**Supplementary Table 4**). When we performed a comparative analysis between NGS and Sanger sequencing using 19 glioma specimens, we found no discrepancies between the two methods.

### Identification of Actionable Alterations Employing the Glio-DNA Panel

The Glio-DNA panel was designed to gather maximum information from a single molecular investigation. To this end, our custom panel includes several druggable genetic alterations linked to tumorigenesis and disease progression. Among clinically actionable genes, we focused on receptor tyrosine kinases (RTKs) (*EGFR*, *KIT, MET* and *PDGFRA*), RTK downstream signaling pathways (*BRAF*, *PIK3CA*, *PIK3R1, RAS*) and genes involved in cell cycle regulation (*CDK4*, *CDK6*, *MDM4* and *RB1*).

**Figure 6:**
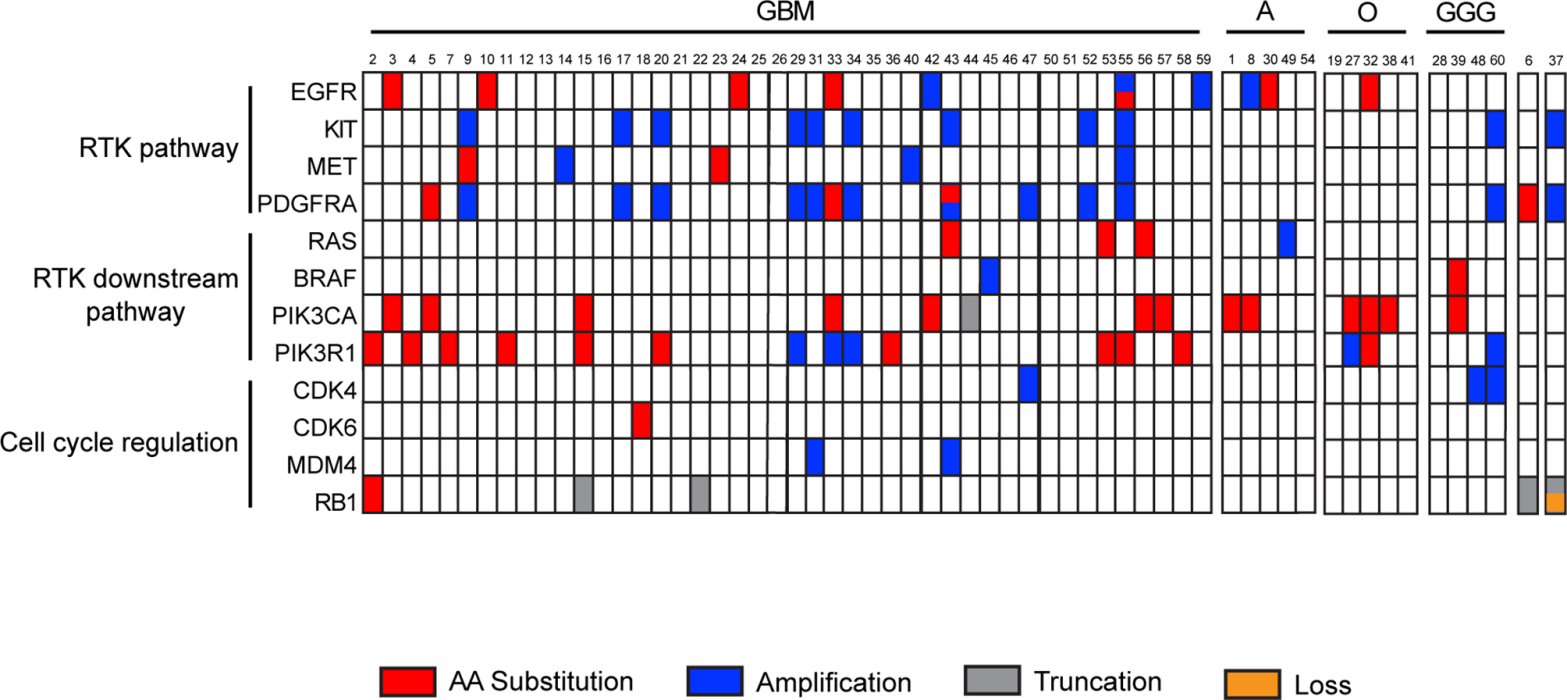
Landscape of recurrent actionable alterations in malignant gliomas. Summary of investigated actionable mutated genes detected by the Glio-DNA panel. Samples are subdivided into groups dependent on diagnostic profile. Single amino acid substitutions are shown in red, truncations in grey, copy number amplifications or deletions in blue and orange, respectively. Sample 6 is a case of gliosarcoma while sample 37 is a case of diffuse midline glioma. GBM, glioblastoma; A, astrocytoma; O, oligodendroglioma; GGG, ganglioglioma; RTK, receptor tyrosine kinase.

We identified *EGFR* mutations (SNVs or CNAs) in 10 of 60 (16.7%) analyzed samples, *KIT* amplifications in 11/60 patients (18.3%), *MET* alterations in 5/60 (8.3%) individuals and *PDGFRA* mutations or amplifications in 15/60 cases (25%). Among RTKs downstream effectors, we found that, 3.3% (2/60) of cases showed *BRAF* mutations, 23.3% (14/60) displayed *PIK3CA* substitutions, 26.7% (16/60) alterations in *PIK3R1* and 6.7% (4/60) *RAS* mutations. Finally, alterations in cell cycle regulation genes were infrequent: 3/60 (5%) patients harbored mutations in *CDK4*, 1/60 (1.7%) in *CDK6*, 2/60 (3.3%) in *MDM4* and 5/60 (8.3%) in *RB1* (**Supplementary Table 4** and **Figure 6**). As expected, GBM samples displayed frequent alterations in the PI3K/AKT/mTOR pathway (21/44 - 47.5%) and in *EGFR* (7/44 - 15.9%) and *KIT* (9/44 - 20.4%). Furthermore, *KIT* and *PDGFRA* were co-amplified in 9/9 (100%) of GBM patients showing alterations in these two genes. Patients affected by high grade astrocytoma displayed alterations involving *PIK3CA* (40%), *EGFR* (40%) and *RAS* (20%) as previously reported (39). As expected, no other genes, besides *IDH1*, *ATRX* and *TP53* were mutated in the single case of low-grade astrocytoma (case 54). Oligodendrogliomas showed alterations in *PIK3CA* (3/5) and *PIK3R1* (2/5). As for individuals affected by ganglioglioma, we observed that 25% of them presented alterations involving *BRAF* and *PIK3CA*, while 50% displayed amplification of *CDK4*, *KIT* and *PDGFRA*. Finally, the single case of diffuse midline glioma (case 37) showed a concomitant truncated form and CNA loss of *RB1* and a CNA gain of *KIT* and *PDGFRA*.

## Discussion

The latest edition of the WHO classification for CNS neoplasms reinforced the importance of an integrated histomolecular classification of malignant gliomas (5, 30) that should include histological tumor typing and grading paired with different molecular biomarkers, such as gene mutations, CNAs or gene fusions. Different technologies, such as polymerase chain reaction, IHC, FISH, Sanger sequencing or mass spectrometric genotyping, are currently in use for the identification of a limited number of molecular markers but none of these methods are able to scale up in order to address the increasing number and variety of alterations occurring in hundreds of cancer-related genes, as well as the novel genetic aberrations continuously emerging (17, 40). The advent of NGS enables the concomitant investigation of multiple molecular markers with high specificity and sensitivity.

Different NGS-based approaches for glioma investigation have been previously described emphasizing a diagnostic benefit with the additional value of the identification of actionable genetic alterations (41–44). For the present study, we designed a customized NGS-based panel detecting several diagnostic, prognostic and predictive molecular biomarkers in a series of 60 patients with diffuse gliomas (**Supplementary Figure 3**). The adoption of a reference standard is very important in order to establish the performance of an NGS analysis, especially if performed employing customized panels (45). Hence, the Glio-DNA custom panel was initially validated on a cell-line DNA standard with engineered genetic variants expressed with a known AF. We also employed a reference FFPE cell-line sample to simulate patient tumor tissue. Our data demonstrate a good reproducibility and sensitivity of the Glio-DNA panel with a high correlation between reference AFs and observed AFs.

We next compared NGS and IHC specificity and sensitivity in detecting several mutations and found that concordance varied depending on the genetic substitution investigated. For example, NGS showed a high specificity (98.11%) and sensitivity (100%) in identifying *IDH1* mutations. On the contrary, NGS was less sensitive than IHC in recognizing *ATRX* alterations. This observation has been previously described (46, 47) and is likely explained by the presence of mutations located in the intronic or promoter regions of the gene that are not covered by the designed amplicons. We also found a low concordance between NGS and IHC in detecting *TP53* mutations, with 80% sensitivity and a 90% specificity for both techniques. The discrepancies between the two tests might also be explained by *TP53* sequence alterations located in intronic regions not covered by the primers used for sequencing. However, a more likely explanation relies in the misinterpretation of low or negative TP53 immunostaining in IHC. Indeed, TP53 detection by IHC results in a high percentage of false-negatives as a negative immunostaining is improperly interpreted as equivalent to the expression of a wild-type protein (48, 49), ignoring the fact that several nonsense mutations do not result in TP53 overexpression and will therefore remain undetected. In this scenario, alternative techniques such as NGS will easily resolve the issue.

FISH is currently the golden standard for the evaluation of chromosomal abnormalities. However, this technique will only identify an individual chromosomal alteration per each reaction (50). Unlike FISH, a properly designed NGS panel will generate information on multiple chromosomal alterations in a single determination. Indeed, with our analysis we obtained LOH information on 1p, 19q and chromosome 7 and 10, demonstrating that NGS was as sensitive and specific as FISH. Furthermore, NGS sequencing allowed the identification of a rare GBM displaying a 1p/19q co-deletion. A high concordance was also found for the detection of imbalance/gain of chromosome 7 and loss of chromosome 10, as well as for the detection of *CDKN2A/B* homozygous and hemizygous deletions.

Recognition of potential actionable targets is a critical need for patients with cancer (51, 52). To date, the standard treatment of GBM is based on surgical resection followed by the Stupp protocol (53), but recurrence generally appears within 6-9 months of diagnosis (54). More than 250 different clinical trials for the evaluation of molecularly targeted treatments were carried out in the last 20 years but only a few studies showed encouraging results. Hence, in current clinical practice treatment choice mostly relies on patient age and performance status (55–57). An advantage of the Glio-DNA panel is its potential utility in detecting clinically relevant genomic alterations in different targetable genes. In our study, we focused on altered RTKs genes and their main downstream signaling pathway as well as on genes involved in cell cycle regulation. Overall, 46 patients (76.7% of the total population) had alterations on the 12 investigated genes but only 22 displayed actionable mutations (pathogenic or likely pathogenic) with an AF ranging from 15 to 67% or CNAs that could be potentially exploited for a targeted treatment. Hence, looking at the identified alterations, sequencing data indicate an actionability rate of 37% (22/60) in our cohort. These results are in line with previous evidence in different cohorts of glioma patients, which report a rate of actionability ranging from 18 to 55% (58, 59). Several ongoing clinical trials investigating targeted treatments directed against actionable genetic alterations in newly diagnosed or recurrent GBM are reported in **Table 2**. Furthermore, targeting gene fusions involving *EGFR*, *FGFR, MET* or *NTRK* may soon represent a promising therapeutic option for several types of cancer including malignant glioma (60). Hence, we are designing an RNA-based panel to investigate gene fusions as clinical biomarkers, in order to offer patients a personalized treatment (61).

**Table 2:** Active clinical trials for the targeted treatment of malignant gliomas.

Gene Alteration	Agent	Combination	Identifier Trial
BRAF	Dabrafenib	Trametinib, Hydroxycloroquine	NCT04201457
	Encorafenib	Binimetinib	NCT03973918
	Dabrafenib	–	NCT02465060
CDK4/CDK6	Abemaciclib	LY3214996	NCT04391595
	Ribociclib	Everolimus	NCT03834740
	Abemaciclib	Bevacizumab	NCT04074785
	Palbociclib	–	NCT02465060
	Palbociclib	–	NCT02530320
EGFR	Afatinib or Osimertinib	–	NCT02465060
KRAS	Ulixertinib	–	NCT04566393
MET	Volitinib	–	NCT03598244
	Crizotinib	–	NCT02465060
	APL-101	–	NCT03175224
NRAS	Binimetinib	–	NCT02465060
	Ulixertinib	–	NCT04566393
PIK3CA	Taselisib or Copanlisib	–	NCT02465060
PDGFRA	Crenolanib	–	NCT02626364

In conclusion, our data demonstrates that NGS represents an accurate, sensitive and valid alternative diagnostic tool that could replace multiple tests for the simultaneous identification of a broad range of genomic anomalies that have become a critical requirement for the proper classification of malignant gliomas. Furthermore, the identification of alterations in druggable genes may improve our ability to select appropriate targeted treatments for glioma patients.

## Data Availability Statement

The datasets presented in this study can be found in online repositories. The name of the repository and accession number can be found below: https://www.ncbi.nlm.nih.gov/sra/PRJNA801939.

## Ethics Statement

The study was conducted according to the guidelines of the Declaration of Helsinki, and approved by the Catania 1 Ethics Committee, Santa Sofia 78 street, Catania, Italy (protocol code: 166/2015/PO; 17/12/2015). The studies involving human participants were reviewed and approved by Azienda Ospedaliero Universitaria Policlinico “G. Rodolico - San Marco. The patients/participants provided their written informed consent to participate in this study.

## Author Contributions

Conceptualization and design of the study, ET and MM. Formal analysis, ET, MM, GB, CR, SM, FrG, and MA. Investigation and data curation, ET, GB, CR, RA, FC, and GMVB. Writing - original draft preparation, ET, MM, GB, GMo, SM, and FB. Writing - review and editing, ET, GB, CR, FB, LM, FC, RA, and PV. Supervision, RC, GMa, FeG, and PV. All authors contributed to manuscript revision, read and approved the submitted version.

## Funding

This study was partially funded by the Research Plan of the University of Catania PIACERI - linea di intervento 2 - entitled (MultiDisciplinar RESEarch and Targeted Therapy for malignant GLIOmas (MD_RESETT_GLIO) and Piano Sanitario Nazionale 2015, Linea Progettuale 6 - Azione 6.3.

## Conflict of Interest

The authors declare that the research was conducted in the absence of any commercial or financial relationships that could be construed as a potential conflict of interest.

## Publisher’s Note

All claims expressed in this article are solely those of the authors and do not necessarily represent those of their affiliated organizations, or those of the publisher, the editors and the reviewers. Any product that may be evaluated in this article, or claim that may be made by its manufacturer, is not guaranteed or endorsed by the publisher.

## Supplementary Material

Supplementary Figure 1 CDKN2A FISH in GBM samples. Representative images of GBM cases showing heterozygous (left panel), homozygous (middle panel) deletion of *CDKN2A* or normal *CDKN2A* (right panel) as assessed by FISH. Red dots represent LSI CDKN2A (p16) signal while green dots represent the CEP 9 signal.

Supplementary Figure 2 Detection of mutant H3-3A and TP53 by immunohistochemistry and NGS. **(A)** Strong and diffuse nuclear immunoreactivity for H3.3K27M (immunoperoxidase; original magnification 200x) (left panel) and corresponding *H3-3A* c.83A>T (p. K27M) mutation detected by NGS (right panel) in the case of a diffuse midline glioma. Results were viewed in the IGV. **(B)** Strong and diffuse nuclear expression of p53 in a *TP53*-mutant glioma (immunoperoxidase; original magnification 200x) (left panel) and corresponding *TP53* c.659A>G (p. Y220C) mutation detected by NGS (right panel) in a case of GBM. Results were viewed in the IGV. **(C)** Focal nuclear immunoreactivity for p53 in a *TP53* wild-type glioma (immunoperoxidase; original magnification 200x). Note that *TP53* is a negative-sense gene relative to the genomic reference sequence. Hence, a nucleotide change is displayed as a reverse complement.

Supplementary Figure 3 Genomic alteration identified in analyzed diffuse gliomas Summary of genomic alterations detected by the Glio-DNA panel and investigated. Samples are subdivided into groups dependent on diagnostic profile. Single amino acid substitutions are shown in red, truncations in grey, copy number amplifications or deletions in blue and orange, respectively. Sample 6 is a case of gliosarcoma while sample 37 is a case of diffuse midline glioma. The letter “X” indicates not available data. GBM: glioblastoma; A: astrocytoma; O: oligodendroglioma; GGG: ganglioglioma.
