# Supplementary figures and images for: A Custom DNA-Based NGS Panel for the Molecular Characterization of Patients With Diffuse Gliomas: Diagnostic and Therapeutic Applications
Source: Front Oncol. 2022 Mar 17;12:861078. doi: 10.3389/fonc.2022.861078 (PMC8969903; doi:10.3389/fonc.2022.861078)

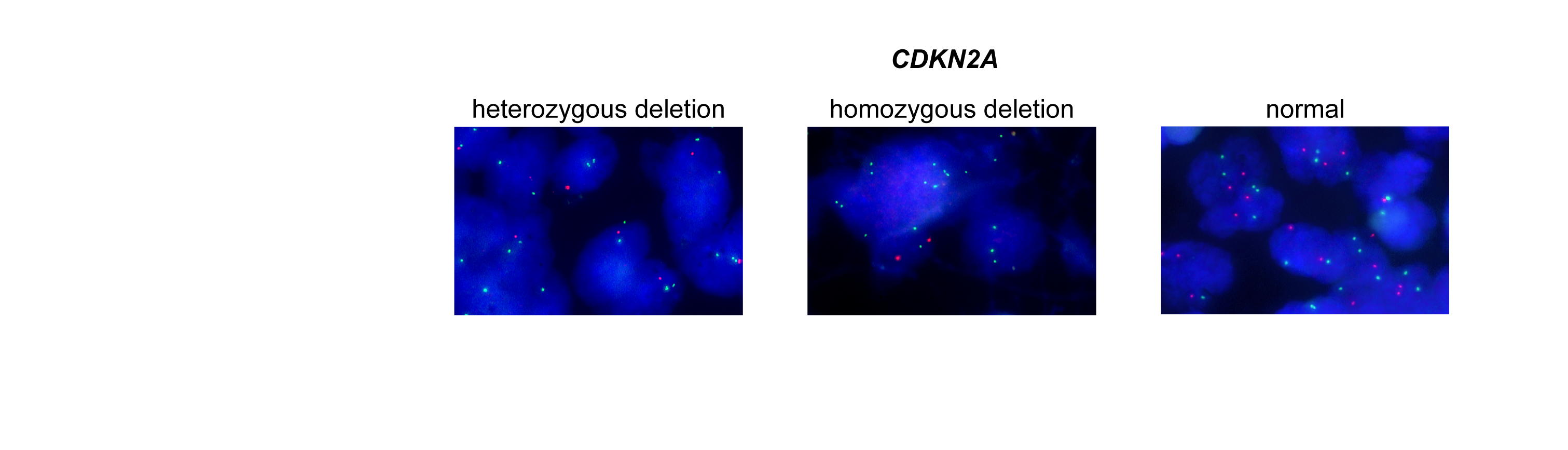

Supplement: Supplementary Figure 1 — CDKN2A FISH in GBM samples. Representative images of GBM cases showing heterozygous (left panel), homozygous (middle panel) deletion of CDKN2A or normal CDKN2A (right panel) as assessed by FISH. Red dots represent LSI CDKN2A (p16) signal while green dots represent the CEP 9 signal. [file Image_1.tif]

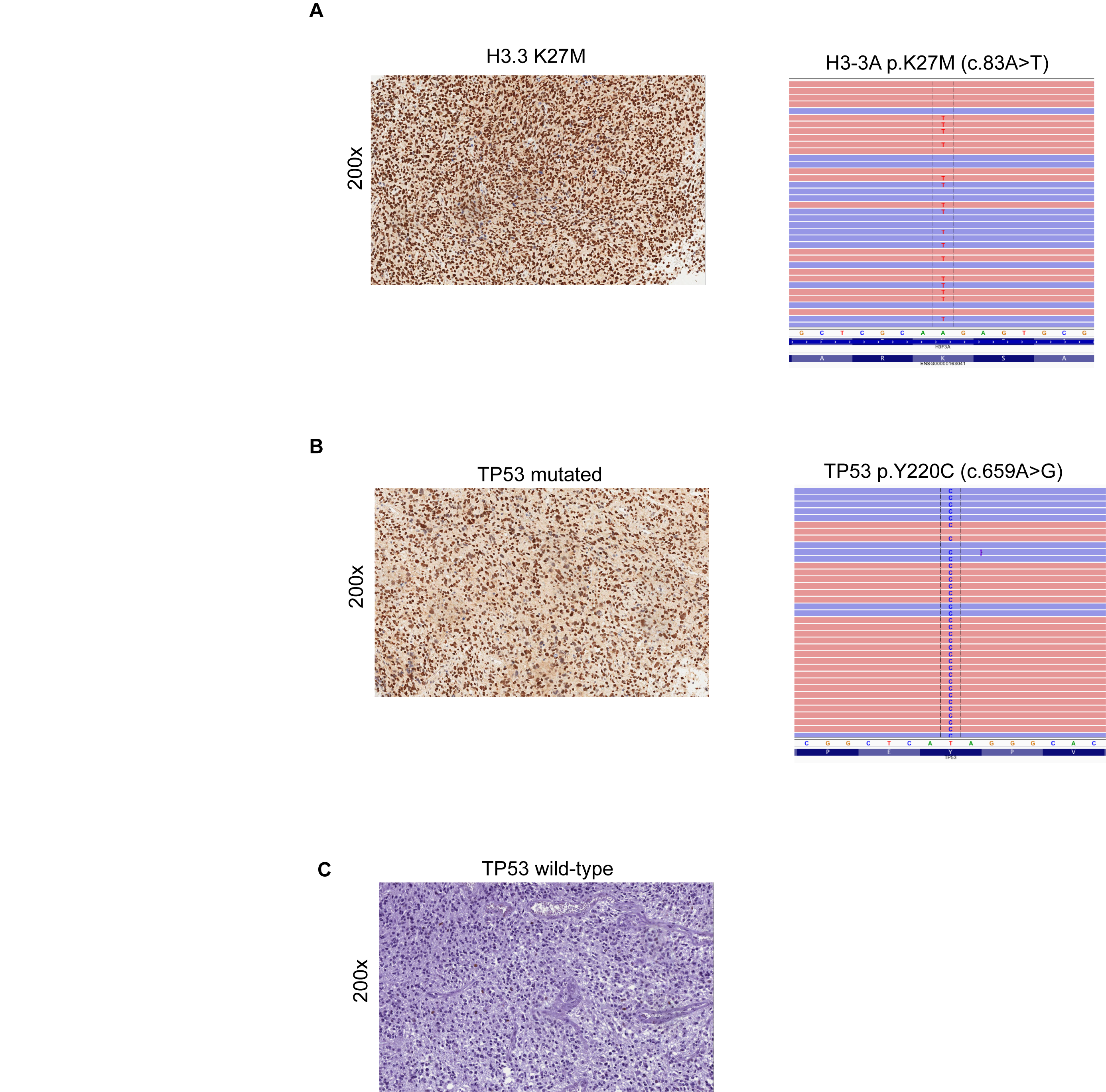

Supplement: Supplementary Figure 2 — Detection of mutant H3-3A and TP53 by immunohistochemistry and NGS. (A) Strong and diffuse nuclear immunoreactivity for H3.3K27M (immunoperoxidase; original magnification 200x) (left panel) and corresponding H3-3A c.83A>T (p. K27M) mutation detected by NGS (right panel) in the case of a diffuse midline glioma. Results were viewed in the IGV. (B) Strong and diffuse nuclear expression of p53 in a TP53-mutant glioma (immunoperoxidase; original magnification 200x) (left panel) and corresponding TP53 c.659A>G (p. Y220C) mutation detected by NGS (right panel) in a case of GBM. Results were viewed in the IGV. (C) Focal nuclear immunoreactivity for p53 in a TP53 wild-type glioma (immunoperoxidase; original magnification 200x). Note that TP53 is a negative-sense gene relative to the genomic reference sequence. Hence, a nucleotide change is displayed as a reverse complement. [file Image_2.tif]

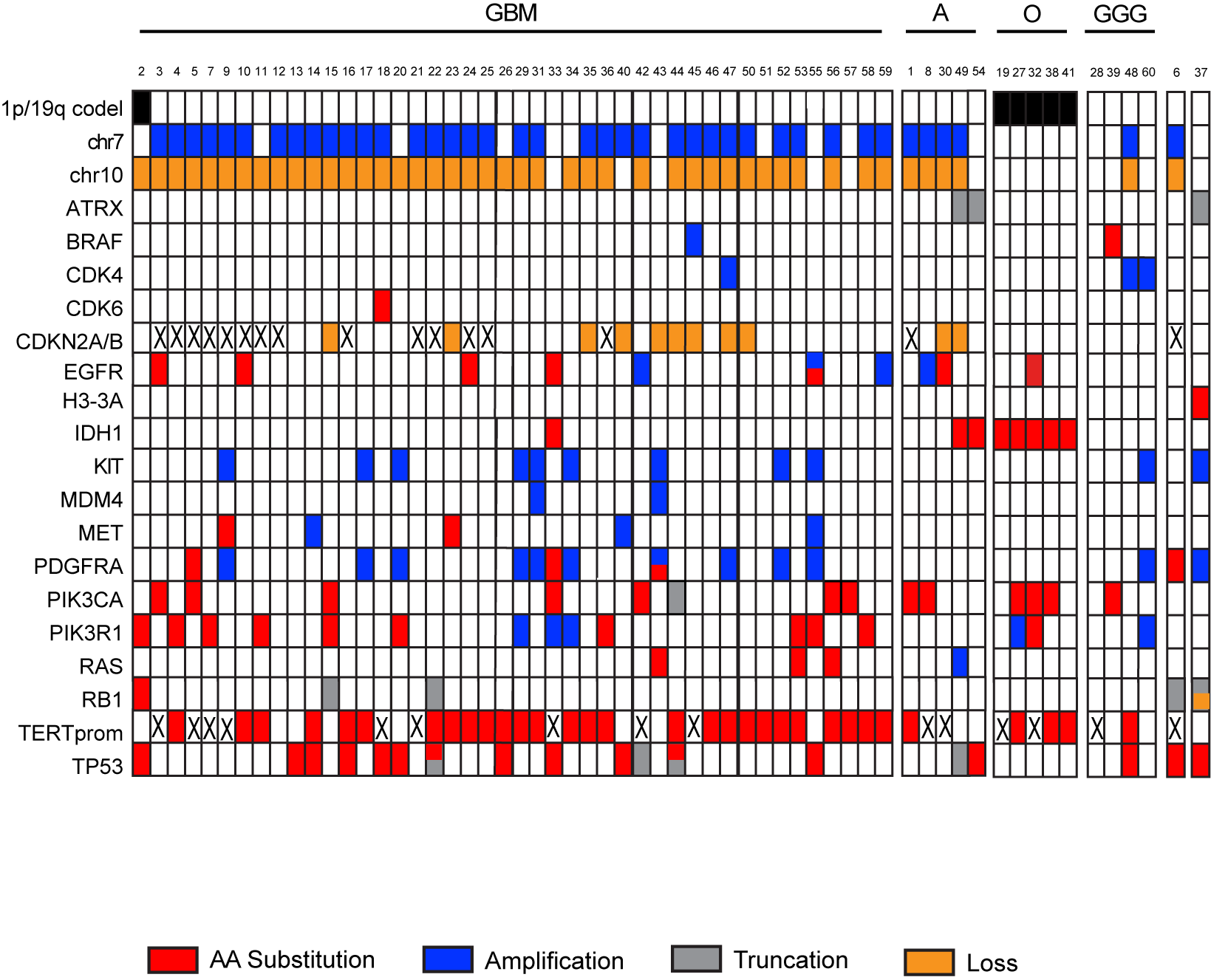

Supplement: Supplementary Figure 3 — Genomic alteration identified in analyzed diffuse gliomas Summary of genomic alterations detected by the Glio-DNA panel and investigated. Samples are subdivided into groups dependent on diagnostic profile. Single amino acid substitutions are shown in red, truncations in grey, copy number amplifications or deletions in blue and orange, respectively. Sample 6 is a case of gliosarcoma while sample 37 is a case of diffuse midline glioma. The letter “X” indicates not available data. GBM: glioblastoma; A: astrocytoma; O: oligodendroglioma; GGG: ganglioglioma. [file Image_3.tif]
