# Supplementary material for: A Custom DNA-Based NGS Panel for the Molecular Characterization of Patients With Diffuse Gliomas: Diagnostic and Therapeutic Applications
Source: Front Oncol. 2022 Mar 17;12:861078. doi: 10.3389/fonc.2022.861078 (PMC8969903; doi:10.3389/fonc.2022.861078)
Supplement: Supplementary file 4 [file Table_1.docx]

Supplemental Table 1: Genes included in the Glio-DNA panel

| ACVR1 | AKT1 | ASCL1 | ATRX | BRAF | CDK4 |
| --- | --- | --- | --- | --- | --- |
| CDK6 | CDKN2A | CDKN2B | CDKN2C | CIC | CTNNB1 |
| DAXX | DDX3X | EGFR | EPCAM | FGFR1 | FGFR2 |
| FGFR3 | FUBP1 | H3-3A | HRAS | IDH1 | IDH2 |
| KIAA1549 | KIT | KLF4 | KRAS | LTBP4 | LZTR1 |
| MDM2 | MDM4 | MET | MYC | MYCN | MLH1 |
| MLH3 | MSH2 | MSH4 | MSH5 | MSH6 | NDRG1 |
| NF1 | NF2 | NFKBIA | NOTCH1 | NRAS | PDGFRA |
| PIK3CA | PIK3R1 | PMS1 | PMS2 | POLD1 | POLE |
| PTCH1 | PTEN | RB1 | SETD2 | SMARCB1 | SMARCA4 |
| SPRED1 | SMO | TACC3 | TERT | TP53 |  |
| MGMT promoter | TERT promoter |  |  |  |  |
