# Supplementary material for: A Custom DNA-Based NGS Panel for the Molecular Characterization of Patients With Diffuse Gliomas: Diagnostic and Therapeutic Applications
Source: Front Oncol. 2022 Mar 17;12:861078. doi: 10.3389/fonc.2022.861078 (PMC8969903; doi:10.3389/fonc.2022.861078)
Supplement: Supplementary file 5 [file Table_2.docx]

Supplemental Table 2: Comparison of variants founds by Glio-DNA panel with variants expected by gDNA reference standard (Horizon HD827- batch 36898)

| **Locus** | **Ref** | **Obs** | **Genes** | **RUN1**  **AF %** | **RUN2**  **AF %** | **AF AVR %** | **AF SD** | **Batch specific NGS AF %** |
| --- | --- | --- | --- | --- | --- | --- | --- | --- |
| chr1:115256530 | G | T | NRAS | 13.25 | 13.63 | 13.44 | 0.269 | 9 |
| chr2:47601106 | T | C | EPCAM | 100.00 | 99.72 | 99.86 | 0.198 | 100 |
| chr2:48018030 | A | G | MSH6 | 35.42 | 33.28 | 34.35 | 1.513 | 36 |
| chr2:48018236 | G | T | MSH6 | 31.74 | 32.90 | 32.32 | 0.820 | 22 |
| chr2:48027921 | T | C | MSH6 | 28.22 | 27.42 | 27.82 | 0.566 | 36 |
| chr3:37056000 | C | A | MLH1 | 21.06 | 23.02 | 22.04 | 1.386 | 15 |
| chr3:41266101 | C | A | CTNNB1 | 30.84 | 34.49 | 32.66 | 2.581 | 25 |
| chr3:41266133 | CCTT | C | CTNNB1 | 10.97 | 11.21 | 11.09 | 0.170 | 8 |
| chr3:178936091 | G | A | PIK3CA | 7.73 | 8.81 | 8.27 | 0.764 | 12 |
| chr3:178947865 | G | A | PIK3CA | 31.97 | 35.69 | 33.83 | 2.630 | 31 |
| chr3:178952085 | A | G | PIK3CA | 19.25 | 17.54 | 18.39 | 1.209 | 20 |
| chr4:1803307 | T | C | FGFR3 | 37.69 | 44.55 | 41.12 | 4.851 | 38 |
| chr4:1803704 | T | C | FGFR3 | 41.03 | 37.34 | 39.18 | 2.609 | 36 |
| chr4:55138600 | G | A | PDGFRA | 36.99 | 31.69 | 34.34 | 3.748 | 36 |
| chr4:55143577 | G | A | PDGFRA | 13.77 | 15.52 | 14.64 | 1.237 | 10 |
| chr4:55152040 | C | T | PDGFRA | 9.48 | 10.11 | 9.79 | 0.445 | 18 |
| chr4:55599321 | A | T | KIT | 10.99 | 12.62 | 11.80 | 1.153 | 10 |
| chr4:55602765 | G | C | KIT | 8.84 | 8.06 | 8.45 | 0.552 | 11 |
| chr4:55604693 | C | A | KIT | 30.31 | 33.25 | 31.78 | 2.079 | 28 |
| chr5:1254594 | C | T | TERT | 30.99 | 27.85 | 29.42 | 2.220 | 32 |
| chr5:1294664 | CG | C | TERT | 28.20 | 29.00 | 28.50 | 0.566 | 31 |
| chr5:67522722 | C | T | PIK3R1 | 16.20 | 18.51 | 17.35 | 1.633 | 23 |
| chr7:6026988 | G | A | PMS2 | 18.81 | 20.48 | 19.64 | 1.181 | 22 |
| chr7:55241707 | G | A | EGFR | 25.06 | 28.57 | 26.81 | 2.482 | 24 |
| chr7:55242464 | TTAAGA | A | EGFR | 0 | 0 | 0 | 0 | 0 |
| chr7:55249063 | G | A | EGFR | 12.13 | 13.37 | 12.75 | 0.877 | 19 |
| chr7:55249071 | C | T | EGFR | 0 | 0 | 0 | 0 | 0 |
| chr7:55259515 | T | G | EGFR | 2.76 | 2.79 | 2.77 | 0.021 | 3 |
| chr7:116339847 | GT | G | MET | 7.50 | 5.48 | 6.49 | 1.428 | 9 |
| chr7:116421967 | T | C | MET | 92.67 | 93.47 | 93.07 | 0.566 | 100 |
| chr7:116436022 | G | A | MET | 5.98 | 5.51 | 5.74 | 0.332 | 5 |
| chr7:128845277 | G | C | SMO | 95.16 | 99.12 | 97.14 | 2.800 | 100 |
| chr7:128852003 | AC | A | SMO | 43.00 | 44.10 | 43.55 | 0.778 | 47 |
| chr7:140453136 | A | T | BRAF | 9.87 | 9.07 | 9.47 | 0.566 | 6 |
| chr7:140494209 | G | A | BRAF | 20.21 | 19.76 | 19.98 | 0.318 | 19 |
| chr8:38275434 | C | A | FGFR1 | 32.99 | 32.11 | 32.55 | 0.622 | 36 |
| chr9:98209594 | G | A | PTCH1 | 48.82 | 45.91 | 47.36 | 2.058 | 51 |
| chr9:98211548 | TG | T | PTCH1 | 33.90 | 36.00 | 34.95 | 1.485 | 29 |
| chr9:98278975 | C | T | PTCH1 | 29.44 | 27.60 | 28.52 | 1.301 | 26 |
| chr9:139391636 | G | A | NOTCH1 | 22.34 | 24.84 | 23.59 | 1.768 | 25 |
| chr9:139396746 | C | T | NOTCH1 | 29.15 | 29.86 | 29.50 | 0.502 | 32 |
| chr9:139397707 | G | A | NOTCH1 | 23.17 | 21.89 | 22.53 | 0.905 | 22 |
| chr9:139407932 | A | G | NOTCH1 | 36.34 | 37.43 | 36.85 | 0.771 | 36 |
| chr9:139409754 | G | A | NOTCH1 | 33.77 | 27.81 | 30.79 | 4.214 | 32 |
| chr9:139418260 | A | G | NOTCH1 | 36.87 | 41.76 | 39.31 | 3.458 | 43 |
| chr11:533328 | C | T | HRAS | 36.02 | 25.75 | 30.88 | 7.262 | 28 |
| chr12:25362777 | A | G | KRAS | 32.05 | 30.30 | 31.17 | 1.237 | 29 |
| chr12:25398281 | C | T | KRAS | 14.09 | 14.70 | 14.39 | 0.431 | 13 |
| chr12:25398284 | C | T | KRAS | 6.07 | 5.30 | 5.68 | 0.544 | 4 |
| chr13:48916887 | A | G | RB1 | 29.84 | 29.46 | 29.65 | 0.269 | 33 |
| chr13:49051481 | T | A | RB1 | 42.23 | 44.82 | 43.52 | 1.831 | 56 |
| chr14:105241378 | C | T | AKT1 | 32.39 | 33.55 | 32.97 | 0.820 | 31 |
| chr17:7579472 | G | C | TP53 | 92.45 | 91.67 | 92.06 | 0.552 | 91 |
| chr17:29553485 | G | A | NF1 | 40.30 | 41.70 | 41.00 | 0.990 | 40 |
| chrX:76938097 | TCT | T | ATRX | 35.67 | 33.48 | 34.57 | 1.549 | 29 |

Ref: reference, Obs: observed, AF: allele frequency, AVR: average, SD: standard deviation
