# Supplementary material for: A Custom DNA-Based NGS Panel for the Molecular Characterization of Patients With Diffuse Gliomas: Diagnostic and Therapeutic Applications
Source: Front Oncol. 2022 Mar 17;12:861078. doi: 10.3389/fonc.2022.861078 (PMC8969903; doi:10.3389/fonc.2022.861078)
Supplement: Supplementary file 6 [file Table_3.docx]

Supplemental Table 3: Comparison of variants founds by Glio-DNA panel with variants expected by FFPE gDNA reference standard (Horizon HD832 - batch 45977)

| **Locus** | **Ref** | **Obs** | **Genes** | **RUN1**  **AF %** | **RUN2**  **AF %** | **AF AVR** | **AF SD** | **Batch specific NGS AF %** |
| --- | --- | --- | --- | --- | --- | --- | --- | --- |
| chr1:115256530 | G | T | NRAS | 9.01 | 10.99 | 10.00 | 1.400 | 8.8 |
| chr1:47601106 | T | C | EPCAM | 100.00 | 100.00 | 100.00 | 0.000 | 100 |
| chr2:48018030 | A | G | MSH6 | 38.79 | 36.54 | 37.67 | 1.591 | 32.7 |
| chr2:48018236 | G | T | MSH6 | 35.26 | 29.81 | 32.54 | 3.854 | 35.9 |
| chr2:48027921 | T | C | MSH6 | 29.18 | 25.58 | 27.38 | 2.546 | 30.7 |
| chr3:37056000 | C | A | MLH1 | 20.90 | 17.77 | 19.34 | 2.213 | 18.1 |
| chr3:41266101 | C | A | CTNNB1 | 35.24 | 33.79 | 34.52 | 1.025 | 28.1 |
| chr3:41266133 | CCTT | C | CTNNB1 | 8.10 | 10.04 | 9.07 | 1.372 | 9.1 |
| chr3:178936091 | G | A | PIK3CA | 7.22 | 11.22 | 9.22 | 2.828 | 10.4 |
| chr3:178947865 | G | A | PIK3CA | 29.04 | 30.59 | 29.82 | 1.096 | 29.9 |
| chr3:178952085 | A | G | PIK3CA | 20.42 | 16.85 | 18.64 | 2.524 | 21.2 |
| chr4:1803307 | T | C | FGFR3 | 37.34 | 37.82 | 37.58 | 0.339 | 38.9 |
| chr4:1803704 | T | C | FGFR3 | 37.41 | 39.02 | 38.22 | 1.138 | 39.8 |
| chr4:55138600 | G | A | PDGFRA | 29.24 | 31.38 | 30.31 | 1.513 | 31.0 |
| chr4:55143577 | G | A | PDGFRA | 15.19 | 16.55 | 15.87 | 0.962 | 13.2 |
| chr4:55152040 | C | T | PDGFRA | 18.28 | 10.77 | 14.53 | 5.310 | 15.3 |
| chr4:55599321 | A | T | KIT | 8.10 | 7.12 | 7.61 | 0.693 | 7.1 |
| chr4:55602765 | G | C | KIT | 8.54 | 8.58 | 8.56 | 0.028 | 10.3 |
| chr4:55604693 | C | A | KIT | 32.38 | 33.80 | 33.09 | 1.004 | 33.1 |
| chr5:1254594 | C | T | TERT | 30.68 | 33.93 | 32.31 | 2.298 | 29.2 |
| chr5:1294664 | CG | C | TERT | 32.30 | 34.30 | 33.30 | 1.414 | 33.2 |
| chr5:67522722 | C | T | PIK3R1 | 16.15 | 18.05 | 17.10 | 1.344 | 15.1 |
| chr7:6026988 | G | A | PMS2 | 18.90 | 18.75 | 18.83 | 0.106 | 20.0 |
| chr7:55241707 | G | A | EGFR | 25.38 | 21.73 | 23.56 | 2.581 | 21.6 |
| chr7:55242464 | TTAAGA | A | EGFR | 0 | 0 | 0 | 0 | 0 |
| chr7:55249063 | G | A | EGFR | 10.62 | 11.35 | 10.99 | 0.516 | 13.1 |
| chr7:55249071 | C | T | EGFR | 0 | 0 | 0 | 0 | 1 |
| chr7:55259515 | T | G | EGFR | 3.60 | 4.56 | 4.08 | 0.679 | 3.8 |
| chr7:116339847 | GT | G | MET | 13.77 | 5.32 | 5.66 | 0.481 | 5.4 |
| chr7:116421967 | T | C | MET | 96.70 | 94.38 | 95.54 | 1.640 | 93.0 |
| chr7:116436022 | G | A | MET | 6.35 | 7.61 | 6.98 | 0.891 | 5.9 |
| chr7:128845277 | G | C | SMO | 99.10 | 97.24 | 98.12 | 1.245 | 100 |
| chr7:128852003 | AC | A | SMO | 47.50 | 50.60 | 49.05 | 2.192 | 47.3 |
| chr7:140453136 | A | T | BRAF | 10.46 | 10.68 | 10.57 | 0.156 | 7.4 |
| chr7:140494209 | G | A | BRAF | 21.12 | 21.40 | 21.26 | 0.198 | 16.5 |
| chr8:38275434 | C | A | FGFR1 | 30.99 | 31.12 | 31.06 | 0.092 | 31.5 |
| chr9:98209594 | G | A | PTCH1 | 47.33 | 46.52 | 46.93 | 0.573 | 48.8 |
| chr9:98211548 | TG | T | PTCH1 | 38.00 | 32.60 | 35.30 | 3.818 | 28.1 |
| chr9:98278975 | C | T | PTCH1 | 28.94 | 28.56 | 28.75 | 0.269 | 34.3 |
| chr9:139391636 | G | A | NOTCH1 | 21.04 | 21.96 | 21.50 | 0.651 | 25.3 |
| chr9:139396746 | C | T | NOTCH1 | 27.74 | 30.40 | 29.07 | 1.881 | 31.4 |
| chr9:139397707 | G | A | NOTCH1 | 22.43 | 24.46 | 23.45 | 1.435 | 26.3 |
| chr9:139407932 | A | G | NOTCH1 | 35.39 | 40.28 | 37.84 | 3.458 | 39.2 |
| chr9:139409754 | G | A | NOTCH1 | 30.17 | 29.04 | 29.61 | 0.799 | 29.1 |
| chr9:139418260 | A | G | NOTCH1 | 41.39 | 36.73 | 39.06 | 3.295 | 38.1 |
| chr11:533328 | C | T | HRAS | 28.46 | 30.07 | 29.27 | 1.138 | 36.8 |
| chr12:25362777 | A | G | KRAS | 32.13 | 33.05 | 32.59 | 0.651 | 30.7 |
| chr12:25398281 | C | T | KRAS | 14.45 | 15.71 | 15.08 | 0.891 | 14.5 |
| chr12:25398284 | C | T | KRAS | 4.15 | 4.21 | 4.18 | 0.042 | 6.6 |
| chr13:48916887 | A | G | RB1 | 26.67 | 31.89 | 29.28 | 3.691 | 32.4 |
| chr13:49051481 | T | A | RB1 | 42.70 | 39.86 | 41.28 | 2.008 | 43.0 |
| chr14:105241378 | C | T | AKT1 | 30.53 | 31.16 | 30.85 | 0.445 | 32.8 |
| chr17:7579472 | G | C | TP53 | 93.00 | 88.89 | 90.95 | 2.906 | 93.3 |
| chr17:29553485 | G | A | NF1 | 37.02 | 37.13 | 37.08 | 0.078 | 40.1 |
| chrX:76938097 | TCT | T | ATRX | 27.07 | 31.13 | 29.10 | 2.871 | 37.2 |

Ref: reference, Obs: observed, AF: allele frequency, AVR: average, SD: standard deviation
